# Potential Applications of Additive Manufacturing in Intervertebral Disc Replacement Using Gyroid Structures with Several Thermoplastic Polyurethane Filaments

**DOI:** 10.3390/biomedicines14020323

**Published:** 2026-01-30

**Authors:** Leandro Hippel, Jan Mussler, Dirk Velten, Bernd Rolauffs, Hagen Schmal, Michael Seidenstuecker

**Affiliations:** 1 G.E.R.N. Center for Tissue Replacement, Regeneration & Neogenesis, Department of Orthopedics and Trauma Surgery, Medical Center-Albert-Ludwigs-University of Freiburg, Faculty of Medicine, Albert-Ludwigs-University of Freiburg, Hugstetter Straße 55, 79106 Freiburg, Germany; leandro.hippel@uniklinik-freiburg.de (L.H.); jan.mussler@uniklinik-freiburg.de (J.M.); bernd.rolauffs@uniklinik-freiburg.de (B.R.); 2 Biomechanics, Offenburg University, Badstraße 24, 77652 Offenburg, Germany; dirk.velten@hs-offenburg.de; 3 Department of Orthopedics and Trauma Surgery, Medical Center-Albert-Ludwigs-University of Freiburg, Faculty of Medicine, Albert-Ludwigs-University of Freiburg, Hugstetter Straße 55, 79106 Freiburg, Germany; hagen.schmal@uniklinik-freiburg.de; 4 Department of Orthopedic Surgery and Traumatology, Odense University Hospital, 5000 Odense, Denmark

**Keywords:** additive manufacturing, 3D-printing, fused deposition modeling FDM, gyroid, intervertebral disc, mechanical properties

## Abstract

**Background**: Intervertebral disc degeneration is a prevalent condition and a major risk factor for disc herniation. Mechanical overload, aging, injury, and disease contribute to the annulus fibrosus’ structural failure, which allows nucleus pulposus material to escape and reduces the capacity to absorb shock. This study builds on previous investigations by evaluating additional thermoplastic polyurethane (TPU) filaments as potential materials for additively manufactured intervertebral disc replacements. **Materials and Methods**: Disc-shaped specimens (Ø50 × 10 mm) were fabricated using fused deposition modeling (FDM). A gyroid infill structure was employed with unit cell sizes ranging from 4 to 10 mm^3^ and wall thicknesses between 0.5 and 1.0 mm. The outer wall thickness varied from 0.4 to 0.8 mm. Four TPU filaments (Extrudr FlexSemiSoft, GEEE-TECH TPU, SUNLU TPU, and OVERTURE TPU) were tested, resulting in 36 parameter combinations per filament. Printed discs were examined via stereomicroscopy. Tensile testing was conducted according to DIN EN ISO 527-1 using Type 5A specimens. Mechanical performance under physiological loading was assessed through uniaxial compression tests, in which samples were compressed to 50% of their height while force–deformation curves were recorded. Target forces were defined as 4000–7500 N to maintain comparability with prior studies. **Results**: Across all filaments, a maximum of three parameter combinations per material achieved forces within the target range. Microscopy confirmed the dimensional accuracy of wall thicknesses with minimal deviation. Tensile strength values for GEEE-TECH, SUNLU, and FlexSemiSoft were comparable (10–11 MPa), while OVERTURE showed significantly lower strength (approximately 9 MPa). Tensile modulus values followed a similar trend: 25–30 MPa for three filaments and 17.5 MPa for OVERTURE. **Conclusions**: All four TPU filaments could be used to fabricate discs that met the mechanical requirements for compression. These results confirm that both the tested TPU materials and gyroid structures are suitable for potential intervertebral disc replacement applications.

## 1. Introduction

Disc degeneration is an increasingly common problem in modern society and often precedes disc protrusion. Causal factors include physical exertion, overuse, the natural aging process, diseases, and injuries. Over time, the fibrous ring of the intervertebral disc develops cracks and small tears, allowing fluid to escape from the gel-like core. This reduces the disc’s ability to absorb shock, which can lead to protrusion or a herniated disc [1]. If conservative treatments are no longer effective, surgical intervention is necessary. The two most important procedures are spinal fusion (spondylodesis), considered the gold standard for treating disc degeneration in the cervical and lumbar spine [1,2,3]. However, spinal fusion has disadvantages, including limited spinal mobility and increased stress on adjacent vertebral segments. This can accelerate the degeneration of adjacent intervertebral discs, a condition known as adjacent segment degeneration (ASD). ASD leads to pain and nerve compression in the affected region [1,2,4,5,6].

A new alternative to spinal fusion is the use of artificial disc prostheses. These prostheses are designed to preserve the spine’s natural range of motion (ROM) while reducing the risk of ASD, as shown in biomechanical evaluations with devices such as ProDisc-C and Prestige discs [7]. In addition, meta-analyses indicate a trend toward lower incidence of adjacent segment degeneration (ASD) and fewer reoperations after cervical disc arthroplasty compared with anterior cervical discectomy and fusion [8]. According to the Federal Statistical Office, the number of spinal fusions and artificial disc implantations performed in Germany increased between 2020 and 2022 [9]. The number of spinal fusions is expected to increase by more than 2000 per year compared to the previous year. This occurred from 67,380 in 2020 to 69,728 in 2021. The number of artificial disc implantations, on the other hand, will remain significantly lower. There were 4415 implantations in 2020, 4205 in 2021, and 3978 in 2022, marking a steady decline. From a biomechanical perspective, the human intervertebral disc must be examined in terms of the annulus fibrosus and the nucleus pulposus because it is not a homogeneous material. Cloyd et al. [10] report E-moduli of 0.1–0.5 MPa for the nucleus pulposus and 4–8 MPa for the annulus fibrosus. In contrast, artificial disc prostheses have an elastic modulus of 100–200 GPa due to their metallic titanium components and polymer components such as polyurethane or polyethylene, which have an elastic modulus between 0.5 and 3 GPa [11]. Therefore, they have no shock-absorbing properties. 

Since 2010, additive manufacturing has been used more and more for medical applications. It can be used to make very precise, custom-made disc prostheses [12]. Various polymeric materials have been explored for this purpose, including flexible polylactic acid (FPLA), which allows for tunable, biomimetic mechanical properties suitable for intervertebral disc tissue engineering [13,14]. These FPLA scaffolds have demonstrated enhanced viscoelasticity and mechanical compatibility with native disc tissue in vitro and in vivo. Similarly, ultra-high molecular weight polyethylene (UHMWPE) has been investigated as a durable structural component for artificial disc replacements, offering favorable wear resistance and improved tensile strength when reinforced with fibers [15]. Such materials demonstrate the potential of polymer-based solutions to bridge the gap between mechanical stability and physiological compliance in intervertebral disc prosthetics. Moreover, the continuous development of additive manufacturing materials expands the range of processable polymers, some of which are now approved for medical use [16].

A previous study [17] investigated whether flexible thermoplastic polyurethane (TPU) filaments could be used to fabricate flexible spinal disc replacements. While that work identified TPU as a highly promising candidate material due to its shock-absorbing properties, the range of printable filaments was limited. In particular, several TPU filaments could not be processed successfully because a drying unit and optimized process parameters were not available at that time. In the present study, this limitation is directly addressed. By implementing an optimized drying protocol at 55 °C before and during printing, along with improved parameter control, it was possible to reliably process those TPU filaments that had previously been unprintable. As a result, the material spectrum investigated here is broader and more representative, allowing for a systematic evaluation of different gyroid geometries and wall designs. Consequently, an extended analysis of additively manufactured TPU gyroid structures with shock-absorbing properties is conducted for their potential application in intervertebral disc replacement. To ensure comparability with the earlier work [17], standardized Ø50 × 10mm intervertebral disc specimens were fabricated using fused deposition modeling (FDM). Consequently, this study can be regarded as a follow-up to the previous study by Gross et al. [17].

## 2. Materials and Methods

### 2.1. Materials

For this study, five different flexible TPU filaments were selected: FlexMed and FlexSemiSoft (extrudr, Lustenau, Austria), SUNLU TPU 95A (SUNLU, Zhuhai, China), OVERTURE TPU (Overture 3D Technologies LLC, Houston, TX, USA), and GEEETECH TPU (HK GETECH Co., Shenzhen, China). To clearly differentiate them during handling, the filaments were used in distinct colors: FlexSemiSoft (transparent), SUNLU (orange), OVERTURE (purple), and GEEETECH (light brown, semi-transparent). An overview of their physical and mechanical properties is given in Table 1.

### 2.2. Methods

#### 2.2.1. Filament Preparation

Before processing, all filaments were conditioned to reduce moisture uptake. Drying was performed in a Creality Space Pi Drying Box (Creality 3D Technology Co., Ltd., Shenzhen, China) at 55 °C for at least five hours. The dried filament was then directly guided via a 2 mm PTFE tube from the drying chamber to the extruder, preventing reabsorption of humidity during printing. Two different printers were used for fabrication: a Prusa MK3S+ (Prusa Research, Prague, Czech Republic) and a Sovol SV04 (Sovol, Shenzhen, China), both of which were not reconstructed apart from exchangeable components, in particular nozzles and the sheet covering the heatbed. Throughout the printing process, the spools remained inside the heated drying chamber.

#### 2.2.2. Three-Dimensional Printing Parameter Optimization

**Figure 1:**
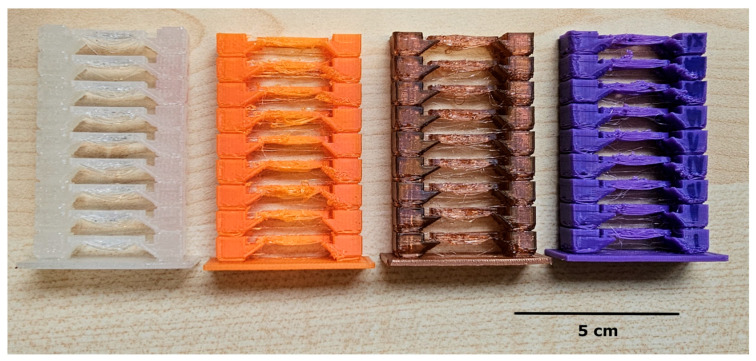
Exemplary illustration of temperature towers made of SemiSoft (transparent), SUNLU (orange), GEEETECH (brown) and OVERTURE (purple) TPU filaments. Temperatures were varied in each step. Printing errors such as stringing are clearly visible at different temperature settings.

To establish suitable process parameters, temperature towers were produced for each filament type to identify the best printing temperature and extrusion speed. Representative examples for FlexSemiSoft, SUNLU, GEEETECH, and OVERTURE are shown in Figure 1. The trials demonstrated that variations in temperature strongly influence surface quality, ranging from stringing effects to incomplete structures. Based on these results, optimized parameter settings were determined. The final values applied in the experiments are highlighted in bold and blue in Table 2.

#### 2.2.3. Additive Manufacturing

Three-dimensional models were designed in Creo Parametric 6.0.6.0 (PTC, Boston, MA, USA) using a construction feature for formula-driven lattices by the cosinusoidal formula known as “black P”-type. To maintain consistency across all experiments while facilitating reproducibility, the gyroid structures were modeled in the form of discs with a diameter of 50 mm and a height of 10 mm [17]. Four Gyroid unit cell sizes (10, 8, 6, and 4 mm) were selected to represent different levels of structural coarseness. Each of these designs was further varied by applying three wall thicknesses (0.5, 0.75, and 1.0 mm) as well as three outer wall thicknesses (0.4, 0.6, and 0.8 mm), resulting in 36 unique design variants (Table A1 in Appendix A). These design variations were realized for each of the four TPU filaments under investigation, yielding a total of 144 disc specimens. The digital models were exported in STL format and subsequently processed in PrusaSlicer 2.7.4 (PRUSA Research, Prague, Czech Republic) for both 3D-printers. Printing was performed on either a Prusa i3 MK3S+ (PRUSA Research) or a SOVOL SV04 printer. Prior to slicing, filament-specific extrusion parameters were adjusted in the software (see Table 2), while other settings such as 100% infill and a constant print speed were applied uniformly. Final G-code files were generated and transferred to the printers. All specimens were printed on a textured spring steel build plate, with adhesion enhanced by a thin layer of glue stick (Kores, Vienna, Austria). The same printing parameters were used for both 3D printers: printing temperature (see Table 2), printing speed of 10 mm/s, first layer 0.1 mm; layer height 0.2 mm and a nozzle size of 0.4 mm. The retract speed was left at the default settings for both printers. In addition to the disc geometries, standardized tensile test specimens (DIN EN ISO 527, Type 5A) were produced from each TPU filament at 100% infill, first layer 0.1 mm; layer height 0.2 mm and a nozzle size of 0.4 mm and 10mm/s printing speed. These samples were reserved for subsequent mechanical characterization.

### 2.3. Sample Characterization

#### 2.3.1. Microscopy

Structural evaluation of the printed specimens was carried out using a stereomicroscope (Olympus SZ61, Olympus Inc., Tokyo, Japan) equipped with an SC30 digital camera. For this purpose, discs were produced at half the height of the standard test specimens, and the printing process was deliberately paused at 50% completion in order to provide unobstructed insight into the internal gyroid geometry. In addition, selected full-height samples were bisected using razor and microtome blades. Dimensional features of the gyroid structures, including wall thickness and pore size, were quantified at multiple locations (≥5 per sample) and compared with the corresponding design specifications from the STL models. Image analysis was performed in ImageJ (FIJI version 1.53t). Scale calibration was established via Stream Motion (Olympus Inc.), which allowed measurements to be reported in metric units rather than pixel lengths. For each variant, at least 20 measurement points were collected per wall type and averaged for subsequent comparison.

#### 2.3.2. Mechanical Properties

To assess filament-specific behavior, tensile testing was performed on dogbone specimens (DIN EN ISO 527, Type 5A) printed from four different TPU filaments (extrudr FlexSemiSoft, OVERTURE TPU 95A, GEETECH TPU 95A and SUNLU TPU 95A). Both the Prusa i3 MK3S+ and the SOVOL SV04 were used to print all of these parts with 100% infill, first layer 0.1 mm; layer height 0.2 mm and a nozzle size of 0.4 mm and 10mm/s printing speed. Testing was carried out on a universal testing machine (LTM10, Zwick-Roell GmbH & Co. KG, Ulm, Germany), with a minimum of five replicates per filament. The mechanical response of the disc-shaped samples was examined in uniaxial compression (Zmart.Pro, Zwick-Roell GmbH & Co. KG). At least three discs per parameter set were tested. Following preload application (1 N), force–displacement data were continuously recorded at a sampling frequency of 1000 Hz while the specimens were compressed at 5 mm/min until 50% strain or a maximum load of 20 kN was reached. The software automatically terminated the test once either limit condition was met. Thickness measurements were taken with a digital caliper at five points before, immediately after, and 24 h post-compression to monitor deformation recovery. Force–displacement curves were averaged from three replicates per condition. Target force ranges for testing were derived from in vivo spinal loading data: vertebral endplates typically withstand 4000–6000 N [18], and intervertebral discs can experience up to 200–250% of body weight during daily activity or exertion [19], corresponding to ~2100 N in an 85 kg individual. To simulate possible peak loads, e.g., during trauma, a broader working window of 4000–7500 N was defined. Samples exceeding 7500 N or compressive strengths above 3.81 MPa (normalized to 50 mm disc diameter) were excluded. This threshold was chosen to reflect the larger contact area of the printed discs relative to native discs, while still aligning with previously reported physiological values (≤3.5 MPa) for intact human lumbar segments [20].

### 2.4. Statistics

All data are reported as mean values accompanied by their respective standard deviations. Unless otherwise stated, we always averaged the measured values of at least 5 samples (DIN EN ISO 527). All measurements were repeated at least 3 times. Prior to statistical testing, the distribution of the data was examined using the Shapiro–Wilk test. For comparisons between groups, a one-way ANOVA with Tukey’s post hoc test was applied, considering differences significant at *p* < 0.05. Statistical processing was carried out with OriginPro 2025 SR1 (OriginLab, Northampton, MA, USA).

## 3. Results

### 3.1. Sample Dimensions

**Figure 2:**
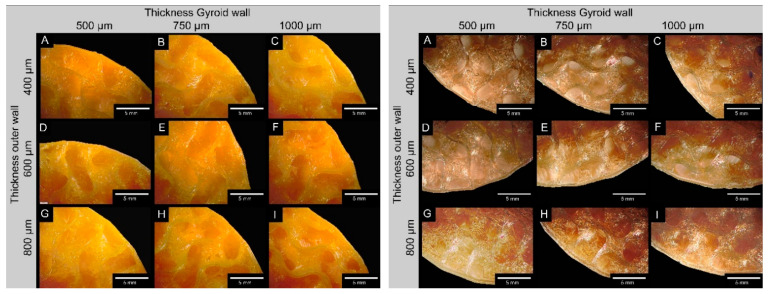
Representative comparison of the varying wall thicknesses of the inner and outer regions of the Gyroid structure made of SUNLU (orange) and GEEETECH TPU (brown). To facilitate accurate wall thickness measurements, 3D printing was paused at 30% to avoid potential measurement errors due to surface curvature (example shown for a 10 mm^3^ Gyroid volume).

**Figure A1:**
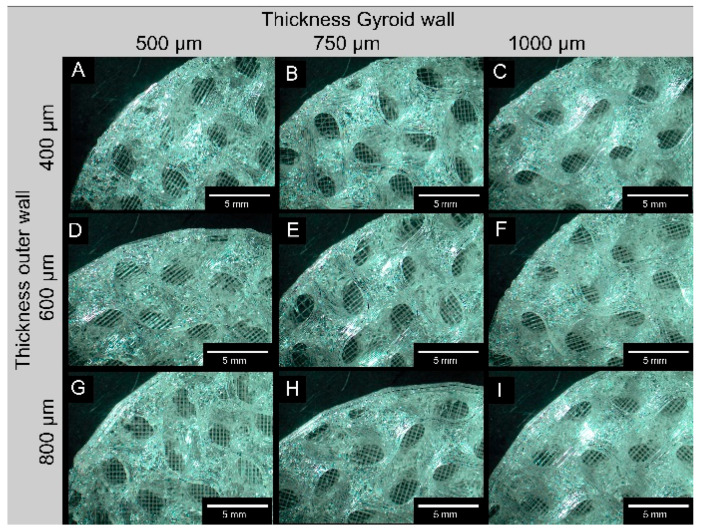
Representative comparison of the varying wall thicknesses of the inner and outer regions of the Gyroid structure made of SemiSoft. To facilitate accurate wall thickness measurements, 3D printing was paused at 30% to avoid potential measurement errors due to surface curvature (example shown for an 8 mm^3^ Gyroid volume).

**Figure A2:**
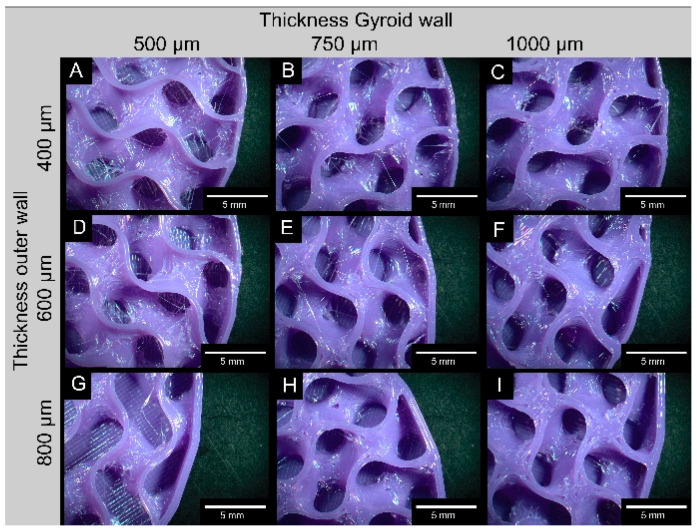
Representative comparison of the varying wall thicknesses of the inner and outer regions of the Gyroid structure made of OVERTURE TPU. To facilitate accurate wall thickness measurements, 3D printing was paused at 30% to avoid potential measurement errors due to surface curvature (example shown for an 8 mm^3^ Gyroid volume).

With the temperatures optimized by the temperature towers (see Table 2), the discs with the different gyroids of varying volumes and wall thicknesses could be printed consistently. In addition, additional drying of the filaments at 55 °C in the filament drying box further improved the printing result. The following Figure 2 shows examples of slices with different wall thicknesses (inner vs. outer) for the different filaments for a Gyroid volume of 8 mm^3^. Due to space limitations, the figures for SemiSoft and Overture can be found in Appendix A (Figure A1 and Figure A2). Table A2 compares the measured wall thicknesses with the target values from the CAD models for the various TPU filaments.

Almost all filaments for the gyroid and the outer wall were within the specified target range, with only a few minor deviations. However, the filaments differ from one another. The largest deviations in the gyroid wall were found with FlexSemiSoft, reaching a maximum of 14%. Overture showed the smallest deviations, reaching a maximum of 2%. Looking at the target/actual comparisons reveals that SUNLU and SemiSoft have the most significant differences (see Table A2). These differences are reflected in the printing errors shown in Figure 2, Figure A1 and Figure A2 in Appendix A. Overture, in particular, showed an increased number of printing errors with lower wall thicknesses but had the fewest target/actual deviations. Further optimizing the printing speed and retraction significantly reduced these errors. However, some dripping and slight stringing were observed. These printing errors were less pronounced with the other filaments.

### 3.2. Mechanical Properties

#### 3.2.1. Tensile Tests

**Figure 3:**
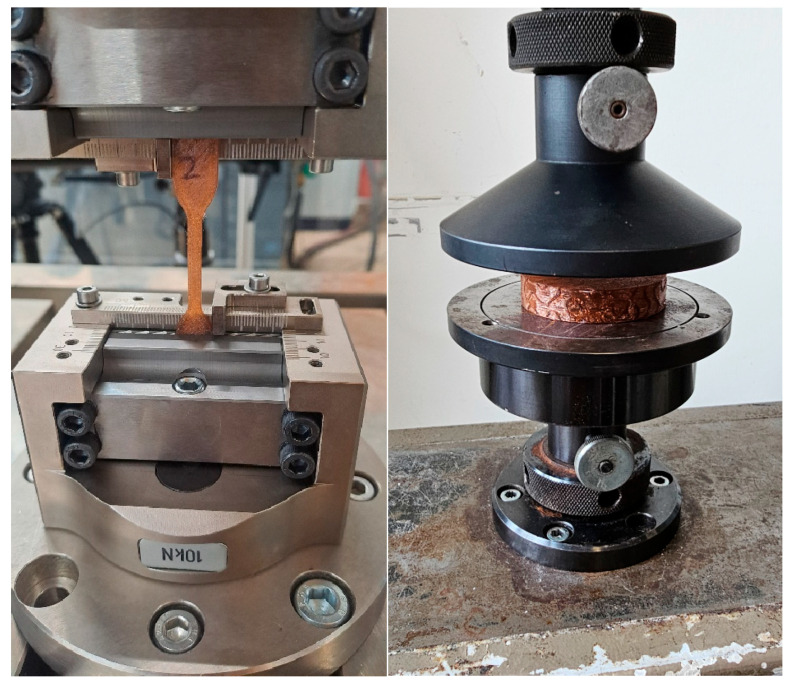
Mechanical Tests on OVERTUE TPU Specimen: (left) Tensile test on specimens 5A (DIN ISO EN 527-2); (right) compression test, *n* = 5.

The tensile tests were carried out in accordance with ISO EN 527 with 5A specimens. The measurements were repeated at least 5 times. Due to the nature of the machine, the samples could only be stretched up to a maximum of 250%. The measurement setup on the Zwick/Roell LTM10 is shown in the following Figure 3. The specimens were pre-tensioned to 5 N and then pulled at a rate of 1 mm/s.

**Figure 4:**
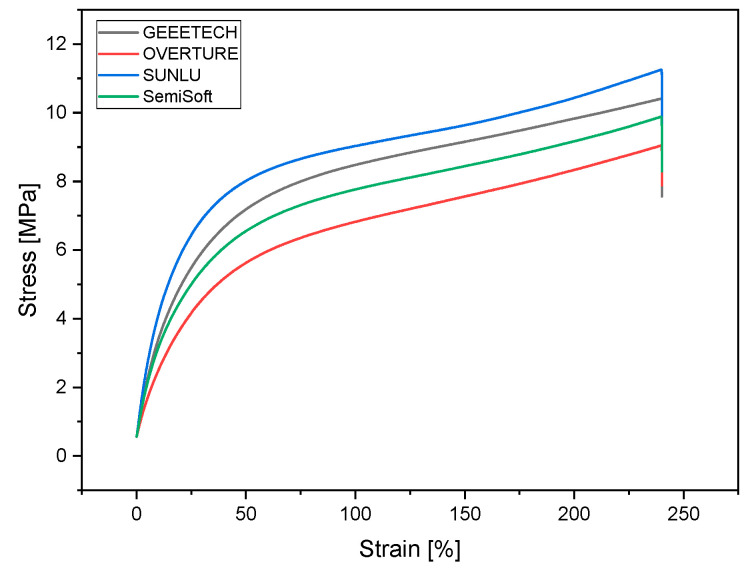
Stress–Strain Curves for the different TPU filaments.

**Figure 5:**
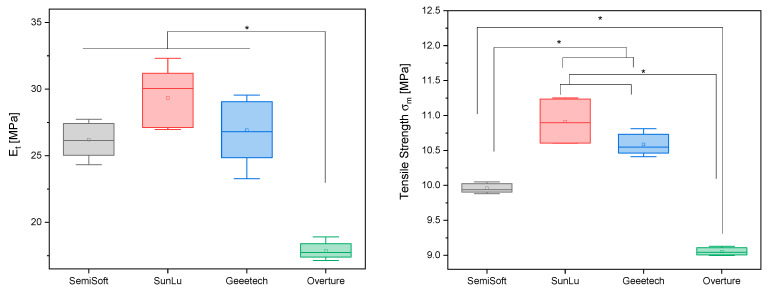
Tensile modulus and tensile strength of the four analyzed filaments; *p* < 0.05 (*); *n* = 5.

The stress–strain curves of the four distinct TPU filaments (GEEETECH, OVERTURE, SUNLU, and SemiSoft) manifest conspicuous viscoelastic behavior, accompanied by elevated ductility (Figure 4). It has been demonstrated that all samples achieve maximum elongations of 250% without brittle fracture, depending on the testing machine. The curves are characterized by an initially steep rise in the elastic range, followed by plastic-viscous flow with moderate stress hardening. GEEETECH and SUNLU demonstrated the highest tensile strengths, at 10.6 ± 0.2 MPa and 10.9 ± 0.15 MPa, respectively, and exhibited substantial hardening with increasing elongation. These materials are well suited for functional applications that require both strength and elastic recovery. In contrast, OVERTURE exhibited the lowest overall load values (9.1 ± 0.05 MPa) and reduced stiffness in the initial range, suggesting a softer material behavior. SemiSoft exhibits a balanced profile between stiffness and elongation capacity, with a reading of 10.0 ± 0.07 MPa. The tensile modulus of SUNLU TPU was the highest of all filaments tested at 29.3 ± 2.2 MPa. The tensile modulus results of Sunlu, Geeetech and SemiSoft were not significantly different. A similar result was found for the compressive strength of the four filaments. Figure 5 and Table 3 summarize the results for tensile modulus and compressive strength.

There is no significant difference between the filaments of SemiSoft, SUNLU, and GEEETECH in terms of tensile modulus (*p* > 0.05). However, OVERTURE’s filament differs significantly from the other three manufacturers in terms of tensile modulus, with *p* < 0.0001. The filaments of SUNLU and GEEETECH do not differ significantly from each other in terms of tensile strength, with *p* = 0.83. But the filament of SemiSoft differs significantly from SUNLU (*p* < 0.01), GEEETECH (*p* < 0.01), and OVERTURE (*p* < 0.001) in terms of tensile strength. The same applies to the significant differences in tensile strength between OVERTURE and SUNLU (*p* < 0.001) and between OVERTURE and GEEETECH (*p* < 0.001).

#### 3.2.2. Compression Tests

##### SemiSoft

**Figure 6:**
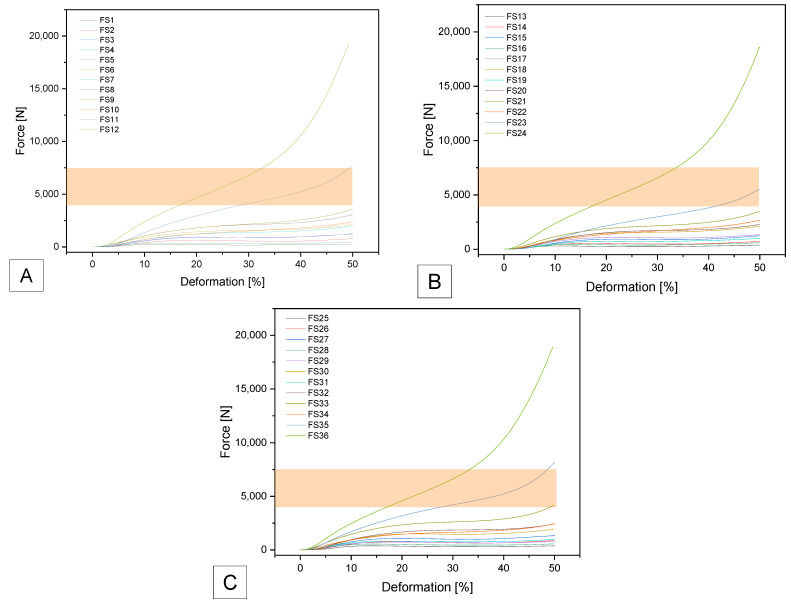
Force–deformation curves for the additively manufactured Gyroid structures with (**A**) 0.4 mm; (**B**) 0.6 mm and (**C**) 0.8 mm outer wall thickness using SemiSoft Filament. The target range is marked as an orange field.

As can be seen in Figure 6, only two samples are within the specified range. These are samples FS23 with F_max_ of 6918 ± 66 N and compressive strength of 3.52 ± 0.03 MPa) and FS33 with F_max_ of 4171 ± 38 N and compressive strength of 2.13 ± 0.02 MPa.

##### SUNLU

**Figure 7:**
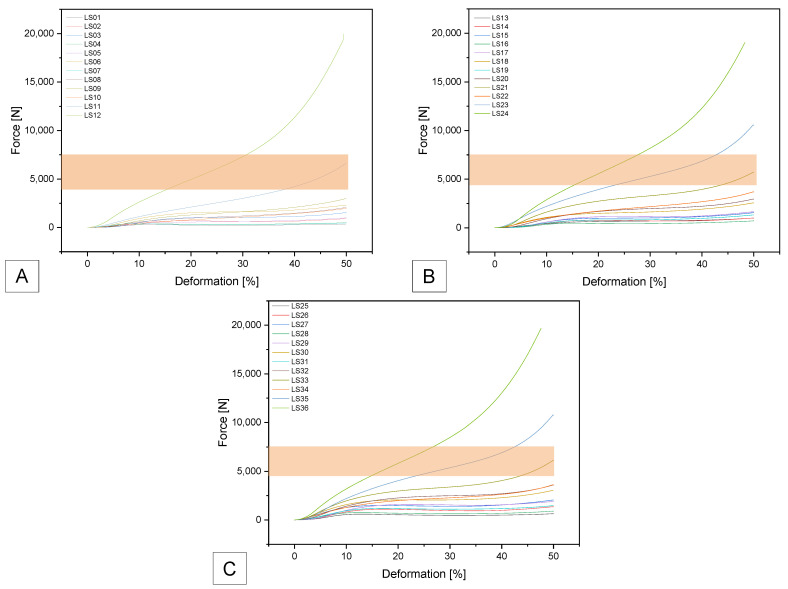
Force–deformation curves for the additively manufactured Gyroid structures with (**A**) 0.4 mm; (**B**) 0.6 mm and (**C**) 0.8 mm outer wall thickness using SUNLU TPU Filament. The target range is marked as an orange field.

For SUNLU filament, three samples: SL11; SL21 and SL33 were within the specified range. SL11 achieved F_max_ of 6607 ± 267 N and compressive strength of 3.4 ± 0.16 MPa; SL21 achieved F_max_ of 5718 ± 222 N and 2.9 ± 0.11 MPa and SL33 achieved F_max_ of 6123 ± 207 N and compressive strength of 3.1 ± 0.08 MPa (see Figure 7).

##### GEEETECH

**Figure 8:**
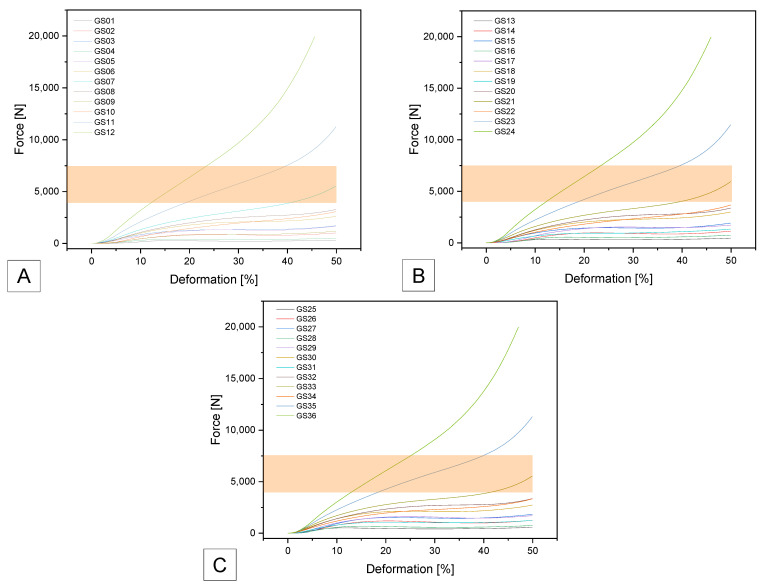
Force–deformation curves for the additively manufactured Gyroid structures with (**A**) 0.4 mm; (**B**) 0.6 mm and (**C**) 0.8 mm outer wall thickness using GEEETECH TPU Filament. The target range is marked as an orange field.

Samples GS07, GS21 and GS33 were within the specification. GS07 had a maximum force of 5533 ± 38 N and a compressive strength of 2.81 ± 0.06 MPa. GS 21 was in a similar range of the maximum tolerated force with 5935 ± 221 N and a compressive strength of 3.02 ± 0.09 MPa. GS33 was also in the same range with 5542 ± 190 N and 2.82 ± 0.08 MPa (see Figure 8).

##### OVERTURE

**Figure 9:**
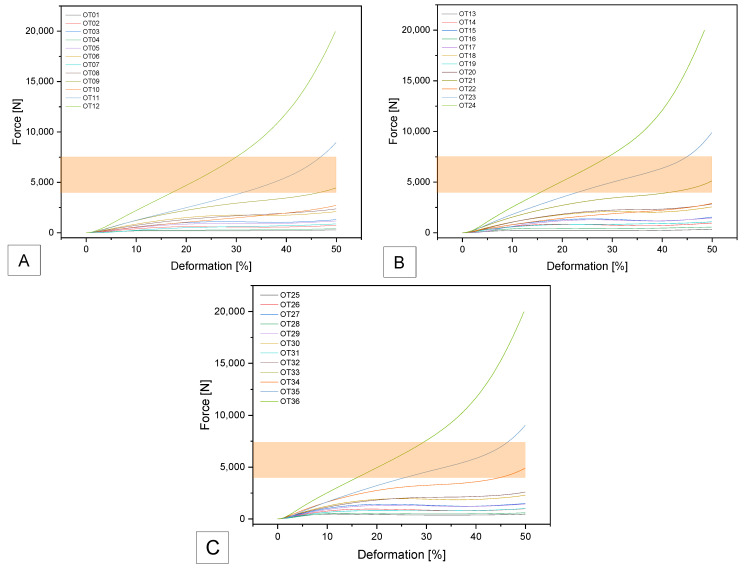
Force–deformation curves for the additively manufactured Gyroid structures with (**A**) 0.4 mm; (**B**) 0.6 mm and (**C**) 0.8 mm outer wall thickness using OVERTURE TPU Filament. The target range is marked as an orange field.

The samples OT09, OT21 and OT33 met the specified requirements. OT09 had a maximum load of 4585 ± 117 N and a compressive strength of 2.29 ± 0.06 MPa. OT21 showed comparable values with a maximum load of 5171 ± 45 N and a compressive strength of 2.55 ± 0.02 MPa. Similarly, OT33 achieved 4922 ± 69 N and 2.38 ± 0.03 MPa (see Figure 9).

The actual values achieved in the maximum tolerated force for the tested samples are summarized in Table 4. The dimensions of all samples that were within the specification are summarized in Table A3 in Appendix A.

#### 3.2.3. Sample Height

**Figure 10:**
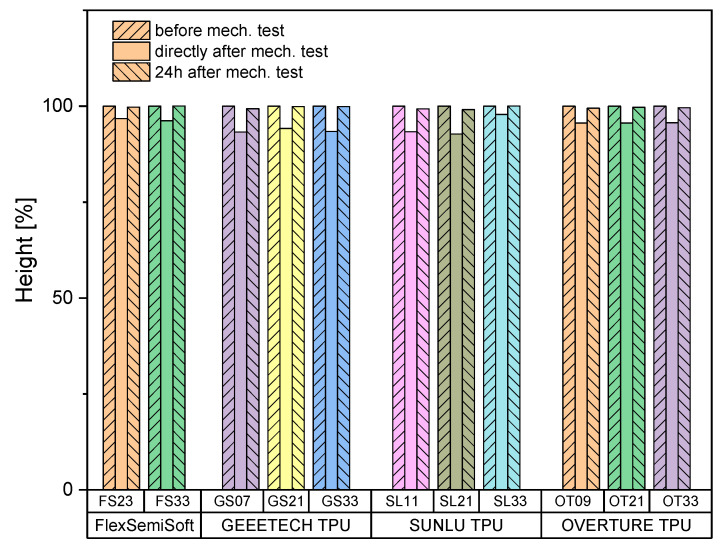
Sample heights before, immediately after, and 24 h after the mechanical test for all samples which were in the target range of 4000–7500 N; *n* = 5.

Sample height was measured using a digital caliper before, immediately after, and 24 h after the mechanical tests. It was found that after compression to 50% of the height, the specimens required a period of time to return to their original height. After 24 h, all specimens had returned to their original height. The following Figure 10 summarize the specimen height distribution for the specimens in the 4000–7500N target range (see Table A3).

## 4. Discussion

Although all of the filaments tested in this work were specified by the manufacturer to have a Shore hardness of 85A or 95A, the filaments differed significantly in their properties in both tensile and compression tests, as well as in their 3D printability. The TPU filaments tested in this work were significantly softer than the FlexMed and FlexHard filaments we had previously tested [17] with a Shore hardness of 95A as specified by the manufacturer. Nevertheless, Sunlu and Geeetech exhibited analogous values for tensile strength and tensile modulus. SemiSoft exhibited a similar trend but demonstrated a substantial disparity in tensile strength. The TPU from Overture exhibited marked differences from the other three filaments in all cases. The tensile strength of SemiSoft was only 1/5 that of FlexHard and 1/3 that of FlexMed from our previous work [17]. A parallel situation was observed for the SUNLU and GEEETECH TPU filaments. In fact, the OVERTURE filament was only 1/10 the strength of FlexHard and 1/6 the strength of FlexMed. Shin et al. [21] described a similarly strong TPU filament with 19.9 MPa, which they also 3D printed using FDM at 180~220 °C. Harynska et al. [22] also printed their TPU for medical applications using FDM, but at 200 °C, achieving a comparable tensile strength of 26 ± 2 MPa. However, Shin et al. [23] printed their TPU as pellets, not filaments, at 190 °C and achieved tensile strengths between 7.42 and 26.93 MPa, depending on the composition. By blending TPU with ABS, Soltanmohammadi et al. [24] achieved compressive strengths in over 150 MPa.

Bayati et al. [25] argue that in extrusion-based 3D printing, the orifice through which the material is extruded is circular, resulting in cylindrical print paths. When these paths are layered and joined together to form the final object, gaps or spaces are often created between the layers [26]. This is illustrated in Figure 2, Figure A1 and Figure A2, which shows a cross-section of a printed part. The size of these spaces, also known as voids, depends on the print quality. Optimal printing parameters and high printability reduce these voids, resulting in a more uniform microstructure of the printed part [27]. Improved overlap, material flow, and wettability minimize voids, thereby improving the mechanical properties of the part [28]. However, the presence of voids can contribute to damage in printed samples. Stresses can develop at the layer interfaces, which can promote crack growth or delamination [26]. In combination with external stresses or environmental influences, these defects can compromise the structural integrity of the printed object, leading to failure or performance degradation over time. This is precisely what we observed initially, before optimizing the print parameters for flexible TPU filaments. The Prusa MK3S+ extruder had difficulty processing the TPU filament with the standard settings, resulting in stringing and dripping on the otherwise flawless prints. However, this issue was resolved by optimizing the print parameters.

Regarding the recovery of the tested discs, we were able to determine, analogous to Feki et al. [29], that our discs behave like intervertebral discs and return to their original thickness overnight. How is this effect achieved? The osmotic pressure of the nucleus pulposus counteracts this stress; however, it is often exceeded during physical activity, resulting in the displacement of water from the disc [30,31].This process leads to a reduction in disc height and volume [32]. During overnight rest, the decrease in external loading allows the osmotic pressure of the nucleus to reabsorb water, facilitating rehydration of the disc [33,34,35].While some research suggests that loss of disc height is due to radial expansion of the annulus fibrosus [36,37]. Botsford et al. [32] argue in their study of diurnal disc changes that such radial bulging is minimal during typical daily activities. They claim that the main factor contributing to the reduction in disc height is the loss of fluid from the disc itself. It is noteworthy that a healthy intervertebral disc can fully recover its original pressure, height, and volume despite being loaded twice as long during the day as during the resting phase at night.

### Limitations

This study has some limitations that should be understood. First, the additively manufactured disc constructs were produced in a simple cylindrical shape rather than an anatomically accurate intervertebral disc shape. This design choice made it so that the results were similar to previous work, but it limits the ability to capture how geometry affects mechanical behavior that may arise in disc replacements that are shaped like an anatomical body part or the human body. Future studies should use realistic disc geometries to better reflect how the discs are actually used and deformed. 

Second, the TPU materials used in this study were not medical-grade filaments. These materials can be used for mechanical testing, but they are not approved for medical use. They may not be as pure, and they may not degrade and break down the same way as medical-grade TPU. So, the results here might not show the full performance of the TPU formulations that are approved for use in clinics.

Third, biocompatibility testing according to ISO 10993 was not conducted. The work was limited to evaluating how well the printed Gyroid structures could resist deformation and how well they could absorb energy. Before we can use this in people, we need to do some tests. These tests will check things like how well it works, if it can cause problems with other things, and how it breaks down over time.

Finally, although this study looked at using TPU filaments with different hardnesses to create mechanical anisotropy, the anisotropy found in native intervertebral discs wasn’t modeled. Future studies should use finite element analysis (FEA) to model and predict how anisotropic behavior depends on material differences, the shape of the structure, and its response to forces. This will make the design simpler and will be like the real mechanical conditions of the intervertebral disc.

## 5. Conclusions

In this study, a test matrix comprising four distinct gyroid sizes, each paired with three distinct wall thicknesses and three outer wall configurations, was evaluated for four TPU filaments. This resulted in a total of 36 samples per filament. By applying an optimized drying protocol at 55 °C prior to and during the printing process, filaments that had been unprintable in a previous study [17] were successfully processed, with substantial reductions in printing errors such as stringing and droplet formation. The TPU filaments with Shore hardness 95A displayed increased flexibility compared to previously reported 95A materials, with 2–3 Gyroid structures per filament achieving peak loads within the target range of 4000–7500 N. These findings indicate that structural variation in Gyroid geometry and wall design can significantly influence the mechanical performance of printed samples. Compared with rigid UHMWPE and titanium implants, the additively manufactured TPU Gyroid structures produced via FDM demonstrate promising shock-absorbing capabilities, reinforcing their potential as intervertebral disc substitutes. Clinically, these results emphasize the importance of tailoring internal architecture to optimize biomechanical properties. Future work should extend to intervertebral disc-shaped specimens with and without outer walls, under both static and dynamic loading conditions, while also accounting for the natural three-dimensional curvature of discs.

## Figures and Tables

**Table 1:** Comparison of the mechanical and physical properties of TPU filaments (manufacturer’s specifications).

Filament	Shore A	Density [g/cm^3^]	Tensile Strength [MPa] *	Elongation at Break [%] **	Tg [°C]	Tm [°C]
SUNLU	95	1.23	21.7	536	−24	190–220
OVERTURE	95	1.18	30.1	332	−23	210
GEEETECH	95	1.30	23.6	525	−24	185–220
SemiSoft	85	1.18	42	550	−24	180–230

* ISO 527-2; ** ASTM D638.

**Table 2:** Temperatures of the different levels of the temperature tower for the TPU filaments used. Arrangement of levels as shown in Figure 1. Final temperatures used for 3D printing are shown in bold.

	Temperature [°C]			
	SemiSoft	Sunlu	Geeetech	Overture
IX	255	210	240	**230**
VIII	252	207	235	227
VII	250	205	**230**	225
VI	247	202	225	222
V	245	200	220	220
IV	242	197	215	217
III	240	195	210	215
II	237	192	205	212
I	**235**	**190**	200	200

Note: The printer bed was preheated to 70 °C for all filaments.

**Table 3:** Summary of the results of the tensile test according to DIN EN ISO 527-1 with a maximum strain of 250% (machine-related); *n* = 5.

**SemiSoft**	**E_t_ [MPa]**	**σ_X_ [MPa]**	**σ_m_ [MPa]**	**ε_m_** **[%]**
Mean	26.2	3.2	10.0	240.6
Min	24.3	3.1	9.9	239.7
Max	27.7	3.2	10.1	241.3
SD	1.3	0.03	0.1	0.6
**SUNLU**	**E_t_ [MPa]**	**σ_X_ [MPa]**	**σ_m_ [MPa]**	**ε_m_** **[%]**
Mean	29.3	3.9	10.9	240.0
Min	27.0	3.6	10.0	239.6
Max	32.3	4.1	11.3	240.8
SD	2.2	0.2	0.2	0.4
**GEEETECH**	**E_t_ [MPa]**	**σ_X_ [MPa]**	**σ_m_ [MPa]**	**ε_m_** **[%]**
Mean	26.9	3.5	10.6	240.7
Min	23.3	3.4	10.4	239.8
Max	29.6	3.6	10.8	241.6
SD	2.4	0.1	0.2	0.7
**OVERTURE**	**E_t_ [MPa]**	**σ_X_ [MPa]**	**σ_m_ [MPa]**	**ε_m_** **[%]**
Mean	17.9	2.5	9.1	239
Min	17.1	2.4	9.0	237.5
Max	18.9	2.5	9.1	239.9
SD	0.6	0.02	0.1	0.9

**Table 4:** Overview of the samples from the different filaments and their outer wall thicknesses that were in the target range of 4000–7500N with their F_max_ and compressive strength (*n* = 3).

**Extrudr FlexSemiSoft**								
**0.4 mm**			**0.6 mm**			**0.8 mm**		
**Sample**	**Fmax [N]**	**σ_D_ [MPa]**	**Sample**	**Fmax [N]**	**σ_D_ [MPa]**	**Sample**	**Fmax [N]**	**σ_D_ [MPa]**
-	-	-	FS23	6918 ± 66	3.52 ± 0.03	FS33	4171 ± 38	2.13 ± 0.02
**SUNLU TPU**								
**0.4 mm**			**0.6 mm**			**0.8 mm**		
**Sample**	**Fmax [N]**	**σ_D_ [MPa]**	**Sample**	**Fmax [N]**	**σ_D_ [MPa]**	**Sample**	**Fmax [N]**	**σ_D_ [MPa]**
SL11	6607 ± 267	3.4 ± 0.16	SL21	5718 ± 222	2.9 ± 0.11	SL33	6123 ± 207	3.1 ± 0.08
**GEEETECH TPU**								
**0.4 mm**			**0.6 mm**			**0.8 mm**		
**Sample**	**Fmax [N]**	**σ_D_ [MPa]**	**Sample**	**Fmax [N]**	**σ_D_ [MPa]**	**Sample**	**Fmax [N]**	**σ_D_ [MPa]**
GS07	5533 ± 38	2.81 ± 0.06	GS21	5935 ± 221	3.02 ± 0.09	GS33	5542 ± 190	2.82 ± 0.08
**OVERTURE TPU**								
**0.4 mm**			**0.6 mm**			**0.8 mm**		
**Sample**	**Fmax [N]**	**σ_D_ [MPa]**	**Sample**	**Fmax [N]**	**σ_D_ [MPa]**	**Sample**	**Fmax [N]**	**σ_D_ [MPa]**
OT09	4585 ± 117	2.29 ± 0.06	OT21	5171 ± 45	2.55 ± 0.02	OT33	4922 ± 69	2.38 ± 0.03

## Data Availability

The data presented in this study are available on request from the corresponding author.

## Abbreviations

ASD	adjacent segment degeneration
CAD	computer-aided design
FDM	fused deposition modelling
FS	Flex Semisoft Filament Sample
G-code	geometry code
GS	Geeetech TPU Filament Sample
OT	Overture TPU Filament Sample
ROM	range of motion
SL	Sunlu TPU Filament Sample
STL	stereo lithography file
TPU	thermoplastic polyurethane
Not listed here are SI abbreviations.	

## Appendix A

**Table A1:** Gyroid Dimensions for additive manufacturing for each Filament.

Sample	Gyroid Dimensions		
	Volume [mm^3^]	Wall Thickness Gyroid [mm]	Wall Thickness Outer Wall [mm]
#01	10	0.5	0.4
#02	10	0.75	0.4
#03	10	1.0	0.4
#04	8	0.5	0.4
#05	8	0.75	0.4
#06	8	1.0	0.4
#07	6	0.5	0.4
#08	6	0.75	0.4
#09	6	1.0	0.4
#10	4	0.5	0.4
#11	4	0.75	0.4
#12	4	1.0	0.4
#13	10	0.5	0.6
#14	10	0.75	0.6
#15	10	1.0	0.6
#16	8	0.5	0.6
#17	8	0.75	0.6
#18	8	1.0	0.6
#19	6	0.5	0.6
#20	6	0.75	0.6
#21	6	1.0	0.6
#22	4	0.5	0.6
#23	4	0.75	0.6
#24	4	1.0	0.6
#25	10	0.5	0.8
#26	10	0.75	0.8
#27	10	1.0	0.8
#28	8	0.5	0.8
#29	8	0.75	0.8
#30	8	1.0	0.8
#31	6	0.5	0.8
#32	6	0.75	0.8
#33	6	1.0	0.8
#34	4	0.5	0.8
#35	4	0.75	0.8
#36	4	1.0	0.8

**Table A2:** Target-performance comparison of the inner and outer walls of the Gyroid for the different filaments, as an example for the Gyroid volume of 10 mm^3^.

Specimen	Gyroid Wall (Target) [mm]	Gyroid Wall (Measured) [mm]	Δ Gyroid Wall [%]	Outer Wall (Target) [mm]	Outer Wall (Measured) [mm]	Δ Outer Wall [%]
FlexSemiSoft						
FS01	0.5	0.49 ± 0.03	2.0 ± 0.06	0.4	0.40 ± 0.02	0.0 ± 0.05
FS02	0.75	0.70 ± 0.02 *	6.7 ± 0.03	0.4	0.41 ± 0.01	2.5 ± 0.03
FS03	1.0	0.90 ± 0.03 *	10.0 ± 0.03	0.4	0.42 ± 0.07	5.0 ± 0.18
FS13	0.5	0.43 ± 0.09	14.0 ± 0.18	0.6	0.61 ± 0.02	1.7 ± 0.03
FS14	0.75	0.70 ± 0.07 *	6.7 ± 0.1	0.6	0.63 ± 0.02	5.0 ± 0.03 *
FS15	1.0	0.99 ± 0.11	1.0 ± 0.11	0.6	0.58 ± 0.04	3.3 ± 0.07 *
FS25	0.5	0.43 ± 0.04 *	14.0 ± 0.08	0.8	0.75 ± 0.05 *	6.3 ± 0.06 *
FS26	0.75	0.72 ± 0.08 *	4.0 ± 0.11	0.8	0.77 ± 0.01 *	3.8 ± 0.01 *
FS27	1.0	0.93 ± 0.05 *	7.0 ± 0.05	0.8	0.74 ± 0.07 *	7.5 ± 0.09 *
SUNLU						
SL01	0.5	0.49 ± 0.03	2.0 ± 0.03	0.4	0.40 ± 0.02	0.0 ± 0.05
SL02	0.75	0.70 ± 0.01 *	6.7 ± 0.01	0.4	0.40 ± 0.01	0.0 ± 0.03
SL03	1	0.92 ± 0.01 *	8.0 ± 0.01	0.4	0.44 ± 0.07	10.0 ± 0.18
SL13	0.5	0.51 ± 0.08	2.0 ± 0.08	0.6	0.62 ± 0.02	3.3 ± 0.03
SL14	0.75	0.72 ± 0.06 *	4.0 ± 0.06	0.6	0.63 ± 0.02 *	5.0 ± 0.08
SL15	1	0.99 ± 0.10	1.0 ± 0.1	0.6	0.56 ± 0.05	6.7 ± 0.06
SL25	0.5	0.45 ± 0.01 *	10.0 ± 0.01	0.8	0.78 ± 0.05 *	2.5 ± 0.02
SL26	0.75	0.74 ± 0.06	1.3 ± 0.06	0.8	0.75 ± 0.07 *	6.3 ± 0.09
SL27	1.0	0.91 ± 0.05 *	9.0 ± 0.05	0.8	0.74 ± 0.01 *	7.5 ± 0.01
GEETECH						
GS01	0.5	0.49 ± 0.01	2.0 ± 0.02	0.4	0.41 ± 0.03	2.5 ± 0.08
GS02	0.75	0.77 ± 0.01 *	2.7 ± 0.01	0.4	0.42 ± 0.02	5.0 ± 0.05 *
GS03	1	1.02 ± 0.06	2.0 ± 0.06	0.4	0.39 ± 0.01	2.5 ± 0.03
GS13	0.5	0.48 ± 0.02 *	4.0 ± 0.04	0.6	0.57 ± 0.02	5.0 ± 0.08
GS14	0.75	0.75 ± 0.01	0.0 ± 0.01	0.6	0.62 ± 0.05	3.3 ± 0.03
GS15	1	0.99 ± 0.05	1.0 ± 0.05	0.6	0.65 ± 0.02	8.3 ± 0.03 *
GS25	0.5	0.50 ± 0.03	0.0 ± 0.06	0.8	0.82 ± 0.02	2.5 ± 0.03
GS26	0.75	0.75 ± 0.02	0.0 ± 0.03	0.8	0.82 ± 0.02	2.5 ± 0.05 *
GS27	1	0.96 ± 0.04	4.0 ± 0.04	0.8	0.81 ± 0.04	1.3 ± 0.05
OVERTURE						
OT01	0.5	0.49 ± 0.02	2.0 ± 0.04	0.4	0.40 ± 0.02	0.0 ± 0.05
OT02	0.75	0.76 ± 0.01	1.3 ± 0.01	0.4	0.41 ± 0.02	2.5 ± 0.05
OT03	1	0.99 ± 0.03	1.0 ± 0.03	0.4	0.40 ± 0.03	0.0 ± 0.08
OT13	0.5	0.49 ± 0.05	2.0 ± 0.10	0.6	0.61 ± 0.02	1.7 ± 0.03
OT14	0.75	0.76 ± 0.03	1.3 ± 0.04	0.6	0.60 ± 0.01	0.0 ± 0.02
OT15	1	1.01 ± 0.04	1.0 ± 0.04	0.6	0.60 ± 0.01	0.0 ± 0.02
OT25	0.5	0.51 ± 0.01	2.0 ± 0.02	0.8	0.81 ± 0.01 *	1.3 ± 0.01
OT26	0.75	0.75 ± 0.01	0.0 ± 0.01	0.8	0.81 ± 0.02	1.3 ± 0.03
OT27	1	0.98 ± 0.03	2.0 ± 0.03	0.8	0.82 ± 0.02 *	2.5 ± 0.03

* significant difference with *p* < 0.05.

**Table A3:** Summary of sample dimensions within the target range of 4000–7500N.

Sample	Gyroid Size [mm]	Gyroid Wall [mm]	Outer Wall [mm]
**Extrudr FlexSemiSoft**			
FS23	4	0.75	0.6
FS33	6	1.0	0.8
**SUNLU TPU**			
SL11	4	0.75	0.4
SL21	6	1.0	0.6
SL33	6	1.0	0.8
**GEEETECH TPU**			
GS07	6	0.5	0.4
GS21	6	1.0	0.6
GS33	6	1.0	0.8
**OVERTURE TPU**			
OT09	6	1.0	0.4
OT21	6	1.0	0.6
OT33	6	1.0	0.8
